# 1-Methyl-3-trifluoro­methyl-5-[(3-chloro­phen­yl)sulfanyl]-1*H*-pyrazole-4-carbaldehyde *O*-(4-chloro­benzo­yl)oxime

**DOI:** 10.1107/S1600536811047970

**Published:** 2011-11-19

**Authors:** Hong Dai, Hai-Jun Zhang, Lei Shi, Kun-Peng Luo, Yu-Jun Shi

**Affiliations:** a College of Chemistry and Chemical Engineering, Nantong University, Nantong 226019, Peoples’ Republic of China

## Abstract

In the title compound, C_19_H_12_Cl_2_F_3_N_3_O_2_S, the 3-chloro­phenyl and 4-chloro­phenyl rings form dihedral angles 89.5 (2) and 11.4 (2)°, respectively, with the pyrazole ring. In the crystal, mol­ecules related by translation along the *a* axis are linked into chains *via* C—H⋯N hydrogen bonds.

## Related literature

For the crystal structure of a related pyrazole oxime studied recently by our group, see: Dai *et al.* (2011 ▶).
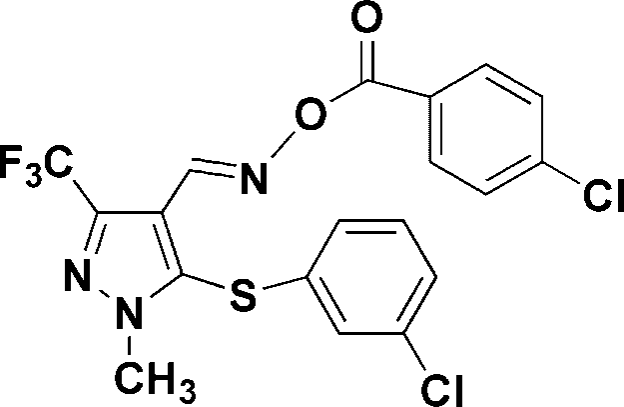


## Experimental

### 

#### Crystal data

C_19_H_12_Cl_2_F_3_N_3_O_2_S
*M* *_r_* = 474.28
Monoclinic, 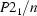

*a* = 8.1405 (16) Å
*b* = 18.680 (4) Å
*c* = 13.737 (3) Å
β = 96.10 (3)°
*V* = 2077.0 (7) Å^3^
*Z* = 4
Mo *K*α radiation
μ = 0.46 mm^−1^
*T* = 113 K
0.18 × 0.16 × 0.12 mm

#### Data collection

Rigaku Saturn CCD area detector diffractometer
Absorption correction: multi-scan (*CrystalClear*; Rigaku, 2008 ▶) *T*_min_ = 0.922, *T*_max_ = 0.947
11802 measured reflections
3654 independent reflections
2794 reflections with *I* > 2σ(*I*)
*R*_int_ = 0.039

#### Refinement

*R*[*F* ^2^ > 2σ(*F* ^2^)] = 0.049
*wR*(*F* ^2^) = 0.137
*S* = 1.10
3654 reflections
273 parameters
H-atom parameters constrained
Δρ_max_ = 0.39 e Å^−3^
Δρ_min_ = −0.38 e Å^−3^

### 

Data collection: *CrystalClear* (Rigaku, 2008 ▶); cell refinement: *CrystalClear*; data reduction: *CrystalClear*; program(s) used to solve structure: *SHELXS97* (Sheldrick, 2008 ▶); program(s) used to refine structure: *SHELXL97* (Sheldrick, 2008 ▶); molecular graphics: *SHELXTL* (Sheldrick, 2008 ▶); software used to prepare material for publication: *SHELXTL*.

## Supplementary Material

Crystal structure: contains datablock(s) r80421a, I. DOI: 10.1107/S1600536811047970/cv5193sup1.cif

Structure factors: contains datablock(s) I. DOI: 10.1107/S1600536811047970/cv5193Isup2.hkl

Supplementary material file. DOI: 10.1107/S1600536811047970/cv5193Isup3.cml

Additional supplementary materials:  crystallographic information; 3D view; checkCIF report

## Figures and Tables

**Table 1:** Hydrogen-bond geometry (Å, °)

*D*—H⋯*A*	*D*—H	H⋯*A*	*D*⋯*A*	*D*—H⋯*A*
C5—H5⋯N2^i^	0.93	2.49	3.365 (4)	156

Symmetry code: (i) 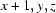.

**Fig. 1.**
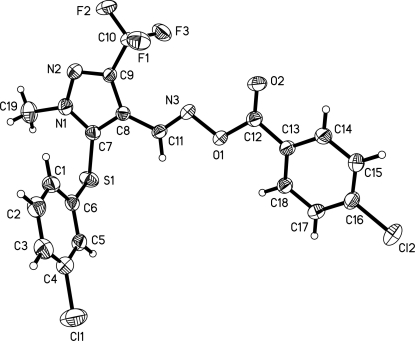
The molecular structure of (I) showing the atomic numbering and 30% probabilty displacement ellipsoids.

## supplementary crystallographic information

### Comment

As a continuation of our structural investigation of pyrazole oxime esters (Dai *et al.*, 2011), we report here the structure of the title compound (I).

In (I) (Fig. 1), all bonds lengths and angles are comparable with those observed in the related compound (Dai *et al.*, 2011). The mean planes p1 (C7/C8/C9/N2/N1), p2 (C1/C2/C3/C4/C5/C6) and p3 (C13/C14/C15/C16/C17/C18) form the following dihedral angles - p2/p1 89.5 (2)°, p3/p1 11.4 (3)°. The crystal packing shows weak intermolecular C—H···N interactions (Table 1), which link molecules into chains along the *a* axis.

### Experimental

To a well stirred solution of 1-methyl-3-trifluoromethyl-5-(3-chlorophenthio)- 1*H*-pyrazole-4-carbaldehyde oxime (4 mmol), and pyridine (5 ml) in 40 ml of dichloromethane, was added dropwise 4-chlorobenzoyl chloride (5 mmol) at room temperature. The resulting solution was heated to reflux for 7 h and cooled to room temperature. The mixture was washed with saturated brine (3 * 30 ml) and dried over anhydrous sodium sulfate. The solvent was evaporated under reduced pressure, the residue was separated by column chromatography on silica gel with petroleum ether/ethyl acetate (12:1 *v*/*v*) as eluent, and then recrystallized from petroleum ether/ethyl acetate acetate to give a colourless crystal.

### Refinement

All H atoms were placed in calculated positions, with C–H = 0.93 - 0.96 Å, and included in the final cycles of refinement using a riding model, with *U*_iso_(H) = 1.2-1.5 *U*_eq_(C).

### Crystal data

**Table:**

C_19_H_12_Cl_2_F_3_N_3_O_2_S	*F*(000) = 960
*M**_r_* = 474.28	*D*_x_ = 1.517 Mg m^−^^3^
Monoclinic, *P*2_1_/*n*	Mo *K*α radiation, λ = 0.71073 Å
Hall symbol: -P 2yn	Cell parameters from 4368 reflections
*a* = 8.1405 (16) Å	θ = 2.2–27.9°
*b* = 18.680 (4) Å	µ = 0.46 mm^−^^1^
*c* = 13.737 (3) Å	*T* = 113 K
β = 96.10 (3)°	Monoclinic, colourless
*V* = 2077.0 (7) Å^3^	0.18 × 0.16 × 0.12 mm
*Z* = 4	

### Data collection

**Table:**

Rigaku Saturn CCD area detector diffractometer	3654 independent reflections
Radiation source: rotating anode	2794 reflections with *I* > 2σ(*I*)
confocal	*R*_int_ = 0.039
Detector resolution: 7.31 pixels mm^-1^	θ_max_ = 25.0°, θ_min_ = 2.2°
ω and φ scans	*h* = −9→9
Absorption correction: multi-scan (*CrystalClear*; Rigaku, 2008)	*k* = −22→22
*T*_min_ = 0.922, *T*_max_ = 0.947	*l* = −14→16
11802 measured reflections	

### Refinement

**Table:**

Refinement on *F*^2^	Secondary atom site location: difference Fourier map
Least-squares matrix: full	Hydrogen site location: inferred from neighbouring sites
*R*[*F*^2^ > 2σ(*F*^2^)] = 0.049	H-atom parameters constrained
*wR*(*F*^2^) = 0.137	*w* = 1/[σ^2^(*F*_o_^2^) + (0.0717*P*)^2^ + 0.2323*P*] where *P* = (*F*_o_^2^ + 2*F*_c_^2^)/3
*S* = 1.10	(Δ/σ)_max_ = 0.001
3654 reflections	Δρ_max_ = 0.39 e Å^−^^3^
273 parameters	Δρ_min_ = −0.38 e Å^−^^3^
0 restraints	Extinction correction: *SHELXL*, Fc^*^=kFc[1+0.001xFc^2^λ^3^/sin(2θ)]^-1/4^
Primary atom site location: structure-invariant direct methods	Extinction coefficient: 0.020 (3)

### Special details

**Table:**

Geometry. All e.s.d.'s (except the e.s.d. in the dihedral angle between two l.s. planes) are estimated using the full covariance matrix. The cell e.s.d.'s are taken into account individually in the estimation of e.s.d.'s in distances, angles and torsion angles; correlations between e.s.d.'s in cell parameters are only used when they are defined by crystal symmetry. An approximate (isotropic) treatment of cell e.s.d.'s is used for estimating e.s.d.'s involving l.s. planes.
Refinement. Refinement of *F*^2^ against ALL reflections. The weighted *R*-factor *wR* and goodness of fit *S* are based on *F*^2^, conventional *R*-factors *R* are based on *F*, with *F* set to zero for negative *F*^2^. The threshold expression of *F*^2^ > σ(*F*^2^) is used only for calculating *R*-factors(gt) *etc*. and is not relevant to the choice of reflections for refinement. *R*-factors based on *F*^2^ are statistically about twice as large as those based on *F*, and *R*- factors based on ALL data will be even larger.

### Fractional atomic coordinates and isotropic or equivalent isotropic displacement parameters (Å^2^)

**Table:**

	*x*	*y*	*z*	*U*_iso_*/*U*_eq_	
Cl1	1.09484 (13)	0.84104 (6)	0.56388 (10)	0.1131 (4)	
Cl2	1.29709 (11)	0.74602 (6)	1.34884 (7)	0.0961 (4)	
S1	0.64859 (10)	1.00969 (4)	0.71200 (6)	0.0663 (3)	
F1	0.1729 (2)	0.85215 (8)	0.92153 (14)	0.0775 (5)	
F2	−0.0064 (2)	0.93357 (11)	0.88380 (14)	0.0867 (6)	
F3	0.1826 (2)	0.94978 (10)	1.00144 (12)	0.0778 (5)	
O1	0.6832 (2)	0.87708 (10)	1.04766 (12)	0.0532 (5)	
O2	0.5304 (3)	0.84250 (12)	1.16752 (17)	0.0833 (7)	
N1	0.3126 (3)	1.00802 (11)	0.71276 (16)	0.0566 (6)	
N2	0.1875 (3)	0.98695 (11)	0.76284 (16)	0.0543 (6)	
N3	0.5309 (3)	0.89838 (12)	0.99338 (15)	0.0538 (6)	
C1	0.5668 (4)	0.88041 (15)	0.6144 (2)	0.0607 (7)	
H1	0.4569	0.8886	0.6238	0.073*	
C2	0.6110 (4)	0.81988 (17)	0.5655 (2)	0.0688 (8)	
H2	0.5297	0.7873	0.5423	0.083*	
C3	0.7709 (4)	0.80690 (17)	0.5504 (2)	0.0715 (8)	
H3	0.7993	0.7657	0.5180	0.086*	
C4	0.8894 (4)	0.85576 (16)	0.5841 (2)	0.0654 (8)	
C5	0.8510 (3)	0.91618 (15)	0.63472 (18)	0.0584 (7)	
H5	0.9336	0.9479	0.6587	0.070*	
C6	0.6885 (3)	0.92892 (14)	0.64942 (16)	0.0508 (6)	
C7	0.4631 (3)	0.98935 (13)	0.75808 (18)	0.0492 (6)	
C8	0.4342 (3)	0.95423 (12)	0.84390 (16)	0.0434 (6)	
C9	0.2602 (3)	0.95408 (12)	0.84192 (16)	0.0433 (6)	
C10	0.1528 (3)	0.92283 (13)	0.91187 (19)	0.0518 (6)	
C11	0.5643 (3)	0.92724 (12)	0.91488 (18)	0.0468 (6)	
H11	0.6739	0.9313	0.9023	0.056*	
C12	0.6607 (3)	0.84774 (13)	1.13649 (19)	0.0513 (6)	
C13	0.8222 (3)	0.82499 (13)	1.18756 (18)	0.0473 (6)	
C14	0.8200 (4)	0.77728 (14)	1.2655 (2)	0.0584 (7)	
H14	0.7197	0.7611	1.2838	0.070*	
C15	0.9663 (4)	0.75393 (14)	1.3154 (2)	0.0629 (8)	
H15	0.9651	0.7220	1.3673	0.076*	
C16	1.1137 (3)	0.77827 (15)	1.2879 (2)	0.0582 (7)	
C17	1.1198 (3)	0.82612 (14)	1.2119 (2)	0.0555 (7)	
H17	1.2206	0.8425	1.1946	0.067*	
C18	0.9727 (3)	0.84924 (13)	1.16177 (19)	0.0517 (6)	
H18	0.9749	0.8814	1.1102	0.062*	
C19	0.2742 (5)	1.0461 (2)	0.6195 (2)	0.0884 (11)	
H19A	0.2762	1.0130	0.5662	0.133*	
H19B	0.3549	1.0830	0.6139	0.133*	
H19C	0.1665	1.0673	0.6176	0.133*	

### Atomic displacement parameters (Å^2^)

**Table:**

	*U*^11^	*U*^22^	*U*^33^	*U*^12^	*U*^13^	*U*^23^
Cl1	0.0650 (6)	0.1302 (9)	0.1451 (10)	0.0138 (5)	0.0163 (6)	−0.0332 (7)
Cl2	0.0651 (6)	0.1262 (8)	0.0921 (6)	0.0249 (5)	−0.0154 (5)	0.0214 (5)
S1	0.0663 (5)	0.0631 (5)	0.0734 (5)	−0.0207 (3)	0.0257 (4)	−0.0077 (3)
F1	0.0803 (13)	0.0574 (10)	0.0992 (13)	−0.0039 (8)	0.0302 (10)	0.0083 (8)
F2	0.0379 (9)	0.1262 (16)	0.0967 (13)	0.0030 (9)	0.0103 (9)	0.0184 (11)
F3	0.0809 (13)	0.0988 (13)	0.0579 (10)	−0.0146 (10)	0.0263 (9)	−0.0142 (9)
O1	0.0370 (10)	0.0717 (11)	0.0500 (9)	0.0044 (8)	0.0002 (8)	0.0088 (8)
O2	0.0458 (13)	0.1175 (18)	0.0892 (15)	0.0122 (11)	0.0195 (12)	0.0396 (13)
N1	0.0603 (15)	0.0591 (13)	0.0498 (12)	0.0021 (11)	0.0025 (11)	0.0099 (10)
N2	0.0485 (13)	0.0582 (12)	0.0553 (13)	0.0069 (10)	0.0016 (11)	0.0036 (10)
N3	0.0374 (12)	0.0684 (14)	0.0536 (12)	0.0051 (10)	−0.0040 (10)	0.0042 (10)
C1	0.0530 (17)	0.0707 (17)	0.0595 (16)	−0.0153 (13)	0.0109 (13)	−0.0025 (13)
C2	0.072 (2)	0.0704 (18)	0.0629 (17)	−0.0216 (16)	0.0033 (16)	−0.0037 (14)
C3	0.080 (2)	0.0724 (19)	0.0627 (18)	−0.0051 (17)	0.0113 (16)	−0.0053 (15)
C4	0.0582 (19)	0.079 (2)	0.0587 (16)	0.0060 (15)	0.0049 (14)	0.0002 (14)
C5	0.0490 (17)	0.0761 (18)	0.0486 (14)	−0.0088 (13)	−0.0013 (12)	0.0014 (13)
C6	0.0522 (16)	0.0591 (15)	0.0413 (13)	−0.0098 (12)	0.0052 (12)	0.0070 (11)
C7	0.0483 (16)	0.0499 (13)	0.0498 (14)	−0.0037 (11)	0.0070 (12)	0.0012 (11)
C8	0.0411 (14)	0.0457 (12)	0.0432 (12)	−0.0016 (10)	0.0038 (10)	−0.0044 (10)
C9	0.0401 (14)	0.0469 (12)	0.0424 (12)	0.0035 (10)	0.0017 (10)	−0.0022 (10)
C10	0.0383 (15)	0.0564 (15)	0.0611 (16)	0.0019 (11)	0.0067 (12)	−0.0012 (12)
C11	0.0360 (13)	0.0532 (14)	0.0509 (14)	−0.0006 (10)	0.0026 (11)	−0.0028 (11)
C12	0.0453 (16)	0.0537 (14)	0.0549 (15)	−0.0002 (11)	0.0050 (13)	0.0065 (11)
C13	0.0425 (15)	0.0488 (13)	0.0505 (13)	0.0023 (11)	0.0048 (11)	0.0007 (11)
C14	0.0511 (16)	0.0626 (16)	0.0623 (16)	0.0010 (13)	0.0105 (13)	0.0150 (13)
C15	0.062 (2)	0.0667 (17)	0.0598 (17)	0.0091 (14)	0.0049 (14)	0.0170 (13)
C16	0.0517 (17)	0.0651 (16)	0.0554 (15)	0.0111 (13)	−0.0057 (13)	0.0009 (13)
C17	0.0418 (15)	0.0617 (16)	0.0627 (16)	−0.0018 (12)	0.0036 (13)	−0.0007 (13)
C18	0.0469 (16)	0.0531 (14)	0.0547 (14)	−0.0015 (11)	0.0032 (12)	0.0072 (11)
C19	0.103 (3)	0.096 (2)	0.0642 (19)	0.011 (2)	0.0001 (18)	0.0349 (18)

### Geometric parameters (Å, °)

**Table:**

Cl1—C4	1.746 (3)	C4—C5	1.379 (4)
Cl2—C16	1.740 (3)	C5—C6	1.380 (4)
S1—C7	1.740 (3)	C5—H5	0.9300
S1—C6	1.783 (3)	C7—C8	1.391 (3)
F1—C10	1.335 (3)	C8—C9	1.414 (3)
F2—C10	1.327 (3)	C8—C11	1.451 (3)
F3—C10	1.328 (3)	C9—C10	1.486 (4)
O1—C12	1.368 (3)	C11—H11	0.9300
O1—N3	1.434 (2)	C12—C13	1.485 (3)
O2—C12	1.189 (3)	C13—C18	1.387 (4)
N1—N2	1.347 (3)	C13—C14	1.394 (4)
N1—C7	1.359 (3)	C14—C15	1.381 (4)
N1—C19	1.469 (3)	C14—H14	0.9300
N2—C9	1.331 (3)	C15—C16	1.373 (4)
N3—C11	1.261 (3)	C15—H15	0.9300
C1—C2	1.382 (4)	C16—C17	1.380 (4)
C1—C6	1.390 (4)	C17—C18	1.385 (4)
C1—H1	0.9300	C17—H17	0.9300
C2—C3	1.361 (4)	C18—H18	0.9300
C2—H2	0.9300	C19—H19A	0.9600
C3—C4	1.372 (4)	C19—H19B	0.9600
C3—H3	0.9300	C19—H19C	0.9600
			
C7—S1—C6	101.54 (12)	F2—C10—F1	106.5 (2)
C12—O1—N3	112.63 (19)	F3—C10—F1	105.9 (2)
N2—N1—C7	112.6 (2)	F2—C10—C9	112.1 (2)
N2—N1—C19	119.0 (3)	F3—C10—C9	112.8 (2)
C7—N1—C19	128.4 (3)	F1—C10—C9	112.2 (2)
C9—N2—N1	104.9 (2)	N3—C11—C8	121.0 (2)
C11—N3—O1	108.1 (2)	N3—C11—H11	119.5
C2—C1—C6	119.3 (3)	C8—C11—H11	119.5
C2—C1—H1	120.4	O2—C12—O1	124.2 (2)
C6—C1—H1	120.4	O2—C12—C13	125.9 (2)
C3—C2—C1	121.5 (3)	O1—C12—C13	109.9 (2)
C3—C2—H2	119.3	C18—C13—C14	119.2 (2)
C1—C2—H2	119.3	C18—C13—C12	123.2 (2)
C2—C3—C4	118.7 (3)	C14—C13—C12	117.6 (2)
C2—C3—H3	120.7	C15—C14—C13	120.2 (3)
C4—C3—H3	120.7	C15—C14—H14	119.9
C3—C4—C5	121.7 (3)	C13—C14—H14	119.9
C3—C4—Cl1	119.5 (3)	C16—C15—C14	119.4 (3)
C5—C4—Cl1	118.8 (2)	C16—C15—H15	120.3
C4—C5—C6	119.1 (3)	C14—C15—H15	120.3
C4—C5—H5	120.4	C15—C16—C17	121.7 (2)
C6—C5—H5	120.4	C15—C16—Cl2	118.9 (2)
C5—C6—C1	119.7 (3)	C17—C16—Cl2	119.4 (2)
C5—C6—S1	116.3 (2)	C16—C17—C18	118.6 (3)
C1—C6—S1	124.0 (2)	C16—C17—H17	120.7
N1—C7—C8	106.5 (2)	C18—C17—H17	120.7
N1—C7—S1	123.51 (19)	C17—C18—C13	120.8 (2)
C8—C7—S1	130.0 (2)	C17—C18—H18	119.6
C7—C8—C9	104.1 (2)	C13—C18—H18	119.6
C7—C8—C11	123.8 (2)	N1—C19—H19A	109.5
C9—C8—C11	132.1 (2)	N1—C19—H19B	109.5
N2—C9—C8	111.9 (2)	H19A—C19—H19B	109.5
N2—C9—C10	117.9 (2)	N1—C19—H19C	109.5
C8—C9—C10	130.2 (2)	H19A—C19—H19C	109.5
F2—C10—F3	106.8 (2)	H19B—C19—H19C	109.5
			
C7—N1—N2—C9	0.0 (3)	C11—C8—C9—N2	178.2 (2)
C19—N1—N2—C9	179.5 (2)	C7—C8—C9—C10	178.2 (2)
C12—O1—N3—C11	177.4 (2)	C11—C8—C9—C10	−2.8 (4)
C6—C1—C2—C3	−0.3 (4)	N2—C9—C10—F2	−0.6 (3)
C1—C2—C3—C4	−0.7 (5)	C8—C9—C10—F2	−179.5 (2)
C2—C3—C4—C5	1.8 (5)	N2—C9—C10—F3	−121.2 (2)
C2—C3—C4—Cl1	−179.0 (2)	C8—C9—C10—F3	59.9 (3)
C3—C4—C5—C6	−2.0 (4)	N2—C9—C10—F1	119.2 (2)
Cl1—C4—C5—C6	178.8 (2)	C8—C9—C10—F1	−59.7 (3)
C4—C5—C6—C1	0.9 (4)	O1—N3—C11—C8	179.75 (19)
C4—C5—C6—S1	−178.0 (2)	C7—C8—C11—N3	177.1 (2)
C2—C1—C6—C5	0.2 (4)	C9—C8—C11—N3	−1.7 (4)
C2—C1—C6—S1	179.0 (2)	N3—O1—C12—O2	−2.5 (4)
C7—S1—C6—C5	−157.1 (2)	N3—O1—C12—C13	178.37 (19)
C7—S1—C6—C1	24.0 (2)	O2—C12—C13—C18	−161.7 (3)
N2—N1—C7—C8	−0.5 (3)	O1—C12—C13—C18	17.4 (3)
C19—N1—C7—C8	−179.9 (3)	O2—C12—C13—C14	17.8 (4)
N2—N1—C7—S1	−179.46 (17)	O1—C12—C13—C14	−163.0 (2)
C19—N1—C7—S1	1.1 (4)	C18—C13—C14—C15	−0.8 (4)
C6—S1—C7—N1	−96.1 (2)	C12—C13—C14—C15	179.7 (3)
C6—S1—C7—C8	85.2 (2)	C13—C14—C15—C16	0.2 (4)
N1—C7—C8—C9	0.7 (2)	C14—C15—C16—C17	0.5 (4)
S1—C7—C8—C9	179.61 (19)	C14—C15—C16—Cl2	−177.8 (2)
N1—C7—C8—C11	−178.3 (2)	C15—C16—C17—C18	−0.7 (4)
S1—C7—C8—C11	0.6 (4)	Cl2—C16—C17—C18	177.6 (2)
N1—N2—C9—C8	0.5 (3)	C16—C17—C18—C13	0.2 (4)
N1—N2—C9—C10	−178.6 (2)	C14—C13—C18—C17	0.6 (4)
C7—C8—C9—N2	−0.8 (3)	C12—C13—C18—C17	−179.9 (2)

### Hydrogen-bond geometry (Å, °)

**Table:**

*D*—H···*A*	*D*—H	H···*A*	*D*···*A*	*D*—H···*A*
C5—H5···N2^i^	0.93	2.49	3.365 (4)	156

Symmetry codes: (i) *x*+1, *y*, *z*.
